# Dengue and malaria coinfection: the first case report in Nepal

**DOI:** 10.1093/omcr/omac022

**Published:** 2022-03-16

**Authors:** Arun Gautam, Ujjwal Aryal, Sudeep Bhandari, Saugat Pradhan, Urza Bhattarai, Akshat Mishra, Sanjib Kumar Sharma

**Affiliations:** Internal Medicine, B.P. Koirala Institute of Health Sciences, Dharan 56700, Nepal; Internal Medicine, B.P. Koirala Institute of Health Sciences, Dharan 56700, Nepal; Internal Medicine, B.P. Koirala Institute of Health Sciences, Dharan 56700, Nepal; Microbiology, B.P. Koirala institute of Health sciences, Dharan 56700, Nepal; Geriatric Medicine, All India Institute of Medical Sciences, New Delhi 110029), India; Internal Medicine, B.P. Koirala Institute of Health Sciences, Dharan 56700, Nepal; Internal Medicine, B.P. Koirala Institute of Health Sciences, Dharan 56700, Nepal

## Abstract

A 21-year-old male from Nepal, with a history of travel to Mumbai 2 months ago, presented with fever with chills and rigors, vomiting and multiple joint pain for 1 week. Clinical examination was noteworthy for tachycardia, hypotension and positive tourniquet test. Lab reports showed NS1-Ag positive, thrombocytopenia, lymphocytosis, transaminesemia, hyperbilirubinemia, increased urea and creatinine. He was treated for severe dengue. His laboratory parameters started improving; however, he had fever with chills and rigors daily and persistent vomiting. Repeat peripheral smear for Malaria showed schizonts and trophozoites of Plasmodium vivax. He recovered following treatment with IV fluids and injection artesunate. The presence of fever even in a critical phase of dengue, the typical rise of temperature daily, and jaundice gave a clue of coinfection with Malaria. On follow-up, after 2 weeks, he had no symptoms, and all the laboratory parameters were normal.

## INTRODUCTION

Dengue and malaria are common vector-borne diseases in Southeast Asia. However, coinfections with these two diseases are less common. Various factors like differences in the vector habitat causing malaria and dengue, the role of immunity in an endemic area, and the fewer chances of concurrence as per the probability principle explain the lesser frequency of coinfections. That is why only a few cases of coinfections are in the reports [1]. Poor access to diagnostic facilities could be next possible reason for nonreporting of such cases in Nepal. Coinfection is the condition when both diseases are present in the same individual at a time. Dengue and malaria coinfection needs a particular concern because; this may increase morbidity and mortality if there are delays in diagnosis and appropriate treatment [2]. The first case report of dengue malaria coinfection was from France in July 2005. Since then, various cases have been reported. Unfortunately, there are very few case reports on dengue and malaria coinfection from many Southeast Asian countries where these two diseases are endemic and compulsory reporting of such cases is recommended [3].

The first case of Dengue was reported from Nepal in 2005, whereas the malaria control program in Nepal was initiated in 1954. As per the Department of Health Services’ annual report, in Nepal for the Fiscal year 2019/20, the total number of *Plasmodium. vivax* cases in 2019/20 was 563. The *P. vivax* malaria in Nepal has decreased in recent years. Still, the spread of cases to different topographical regions of Nepal has a significant challenge in controlling the infection. The number of Dengue cases in Nepal in the Fiscal year 2017/18 was 811, whereas the number in 2019/20 was 10 808. This shows the upsurge of incidence and burden of dengue virus infection in recent years in Nepal [4].

## CASE REPORT

**Figure 1:**
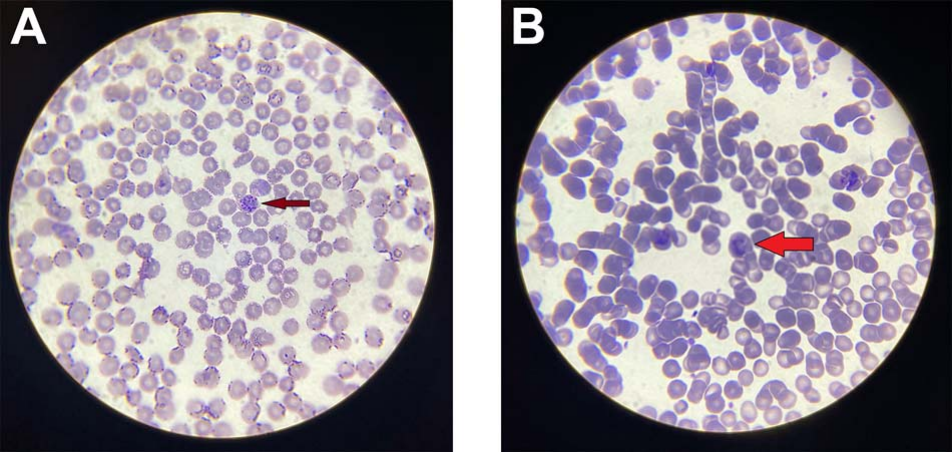
(A) Peripheral blood smear with Giemsa stain; red arrow shows schizont of *P. Vivax* with magnification 1000× (oil immersion). (B) Peripheral blood smear with Giemsa stain; red arrow shows late trophozoite of *P. Vivax* with magnification 1000× (oil immersion).

We report a case of a 21-year-old male from Nepal, a resident of the hilly region of Nepal, with a history of travel to Mumbai, India, 2 months ago. He had not received any prophylaxis therapy for malaria during his travel to Mumbai. He had no history of dengue or malaria infection in the past. He presented with fever with chills and rigors, vomiting on and off, bodyache and multiple joint pain for 1 week. The maximum-recorded temperature was 102°F. He had tachycardia of 110 bpm and BP = 80/50 mm Hg. The tourniquet test was positive. His laboratory reports are shown in Table 1. Positive NS1-Ag, thrombocytopenia, lymphocytosis, transaminesemia, hyperbilirubinemia, anemia, increased urea, creatinine and granular cast on urine routine examination were noteworthy. Ultrasonography abdomen showed spleen of size 12 cm. We started treatment for the severe dengue. The patient became hemodynamically stable with 1 L of bolus normal saline. We continued adequate hydration, and repeated his hemoglobin and hematocrit daily. He was hemodynamically stable; however, his Hemoglobin and Hematocrit was gradually decreasing to 9.6 g/dl and 32.1%, respectively on the fourth day of admission. Other laboratory parameters were improving gradually, however, he was having vomiting of around 5–6 episodes a day and a fever of 102–103°F with chills and rigors daily at around noon. His urine culture and blood culture reports were normal. We could not detect any other focus of infection. We repeated his peripheral smear for Malarial parasite on the third day of admission during the peak of fever and started him on artesunate injection empirically. From the next day, his fever and vomiting subsided. The repeat peripheral smear for the malarial parasite report showed schizonts and trophozoites of *P. vivax* as shown in Fig. 1A and Fig. 1B, respectively. We completed his artesunate dose and started him on primaquine therapy after his G6PD reports were normal. On follow-up after 2 weeks of discharge, he had no symptoms and all the laboratory parameters were within normal range.

**Table 1:** Laboratory parameters

Lab parameters	Day 1	Day 2	Day 3	Day 4
Hb(g/dl)	15.0	12.8	10.5	9.6
PCV(%)	44	37	33.8	32.1
Total leukocyte counts(/μl)	5500	5700	6800	7400
Differential leukocyte count	N43L48M08E01	N42L49M08E01	N75L20M04E01	N76L17M04E03
Platelets(/μl)	16 000	12 000	40 000	92 000
Urea/creatinine(mg/dl)	65/1.5	38/1.1		
Bilirubin: Total/conjugated(mg/dl)	6.8/3.8		5.9/3.4	
ALT/AST/ALP(U/L)	79/110/50		85/123/28	
NS1 Ag/IgG, IgM for dengue	Positive/negative			
RDT for malaria/RDT for scrub typhus/leptospira IgM,IgG	Negative			

## DISCUSSION

Fever in dengue usually subsides after the patient reaches the critical phase [5]. The presence of fever after 7 days of illness, even in a critical phase of dengue, gave us a clue to consider associated infection other than dengue in our case. The typical rise of temperature daily at a similar time, jaundice and anemia gave a clue of coinfection with malaria. The coinfections may get missed because of the similar clinical picture of mono-infection of these two conditions [6]. It has been observed that presence of jaundice in a case of dengue and presence of spontaneous bleeding in a case of malaria suggests coinfection [7].Usually, in acute febrile illness, we often forget to look after the coinfections once one of the conditions is diagnosed [3]. It has been hypothesized that the activation of the latent hypnozoites of *P. vivax* can occur following various systemic illnesses [8]. Therefore, the detection of *P. vivax* along with any other infection in endemic areas of malaria can also be due to the reactivation of such hypnozoites. Detection of *P. vivax* in the later course of illness in our patient could be an example of relapse of *P. vivax* precipitated by dengue according to the ‘activation of latent hypnozoites hypothesis’.

The study of cases with coinfection may help us suspect and identify such entities quickly, thus help to avoid the diagnostic dilemmas. In patients residing in the endemic area of dengue and malaria, or with history of recent travel to such areas, we should also think of dengue malaria coinfection. Therefore, early suspicion, if the clinical picture is not fitting well to the monoinfection, may help in early diagnostic and therapeutic intervention. It may reduce the morbidity and fatality of coinfections.

## References

[1] Wiwanitkit V . Concurrent malaria and dengue infection: a brief summary and comment. Asian Pac J Trop Biomed 2011;1:326–7. 23569785 10.1016/S2221-1691(11)60053-1 PMC3614227

[2] Santana VD, Lavezzo LC, Mondini A, Terzian AC, Bronzoni RV, Rossit AR, et al. Concurrent dengue and malaria in the Amazon region. Rev Soc Bras Med Trop 2010;43:508–11. 21085859 10.1590/s0037-86822010000500007

[3] Sahu PS, Sahu M, Ambu S. A review of concurrent infections of malaria and dengue in Asia. Asian Pac J Trop Biomed 2016;6:633–8.

[4] Government of Nepal, Department of Health Services . Annual Report Department of Health Services 2076/77 (2019/20) [Internet]. Kathmandu (NP): Government of Nepal, Ministry of Health and Population; 2020 [cited 2021 Dec 30]. Availabe from: https://dohs.gov.np/wp-content/uploads/2021/07/DoHS-Annual-Report-FY-2076-77-for-website.pdf

[5] Bhargava A, Ralph R, Chatterjee B, Bottieau E. Assessment and initial management of acute undifferentiated fever in tropical and subtropical regions. BMJ 2018;363:k4766. 30498133 10.1136/bmj.k4766

[6] Abdul-Ghani R, Mahdy MA, Alkubati S, Al-Mikhlafy AA, Alhariri A, Das M, et al. Malaria and dengue in Hodeidah city, Yemen: high proportion of febrile outpatients with dengue or malaria, but low proportion co-infected. PLoS One 2021;16:e0253556. 34170955 10.1371/journal.pone.0253556 PMC8232408

[7] Magalhães BM, Siqueira AM, Alexandre MA, Souza MS, Gimaque JB, Bastos MS et al. P. vivax malaria and dengue fever co-infection: a cross-sectional study in the Brazilian Amazon. PLoS Negl Trop Dis 2014;8:e3239. 25340346 10.1371/journal.pntd.0003239 PMC4207662

[8] Shanks GD, White NJ. The activation of vivax malaria hypnozoites by infectious diseases. Lancet Infect Dis 2013;13:900–6. 23809889 10.1016/S1473-3099(13)70095-1

